# Identification of four serum microRNAs from a genome-wide serum microRNA expression profile as potential non-invasive biomarkers for endometrioid endometrial cancer

**DOI:** 10.3892/ol.2013.1338

**Published:** 2013-05-08

**Authors:** WENHUI JIA, YUANZHE WU, QIN ZHANG, GE GAO, CHENYU ZHANG, YANG XIANG

**Affiliations:** 1 State Key Laboratory of Pharmaceutical Biotechnology, School of Life Sciences, Nanjing University, Nanjing, Jiangsu 210093, P.R. China; 2 Department of Gynaecology and Obstetrics, Jinling Hospital, Nanjing, Jiangsu 210093, P.R. China

**Keywords:** endometrioid endometrial cancer, serum microRNAs, biomarker, diagnosis

## Abstract

Serum microRNAs (miRNAs), with their remarkable stability and unique concentration profiles in patients with various diseases, are promising non-invasive biomarkers for tumor detection. The present study investigated the altered profiles of serum microRNAs in patients with endometrioid endometrial cancer (EEC) in order to predict the malignancy of the disease at a relatively early stage. TaqMan^®^ low-density arrays (TDLAs) were used to perform an analysis in the initial screening phase using serum samples pooled from seven EEC patients and 20 controls. The differential expression was validated using a hydrolysis probe-based stem-loop quantitative reverse transcription polymerase chain reaction (qRT-PCR) in samples taken from 26 EEC patients and 22 age- and gender-matched healthy controls. The data obtained from the TLDAs demonstrated that 22 serum miRNAs were markedly upregulated in the EEC patients compared with the controls. The qRT-PCR analysis further identified a profile of four serum miRNAs (miR-222, miR-223, miR-186 and miR-204) as a fingerprint for EEC detection. The area under the receiver operating characteristic (ROC) curve of this four-serum miRNA signature was 0.927, which was markedly higher than that of carbohydrate antigen 125 (CA-125; 0.673). The four-miRNA signature identified by genome-wide serum miRNA expression profiling analysis provides a novel, non-invasive approach for EEC diagnosis.

## Introduction

Endometrial carcinoma is the most common invasive malignancy of the female genital tract in the Western world, and the fourth most common cancer in the United States of America (1). The incidence has increased steadily during the past three decades (2,3), with endometrioid endometrial cancer (EEC) being the most dominant subtype and accounting for >80% of the total cases (4–7).

With regard to diagnosing EEC, the results of a pelvic examination are usually normal, particularly in the early stages of the disease. A pap test is also insufficient to detect EEC. Although the morphological alterations provide significant insights into EEC, highly sensitive and specific molecular prognostic biomarkers are required to better predict the outcome of EEC. However, the sensitivity and positive predictive value of carbohydrate antigen 125 (CA-125) measurements are relatively low (sensitivity 58.2%, using a cut-off value 35 kU/l) in detecting this malignancy (8). These deficits have created significant interest in the search for novel predictive markers for EEC.

As carcinogenic events may be acquired over a number of years, only adult stem/progenitor cells are believed to have a lifespan that is sufficiently long enough to accumulate the genetic damage necessary to give rise to cancer stem cells (CSC), which are hypothesized to initiate carcinomas (9,10). Research is emerging with regard to the role of microRNAs (miRNAs) in a variety of pathological conditions, including solid and hematological malignancies. miRNAs, a class of small non-coding RNAs (18–25 nucleotides), are known to form an imperfect paring at the 3′-end of the untranslated regions (UTR) of a target locus, resulting in mRNA degradation or translational inhibition (11). It is becoming clear that miRNAs play critical roles in various biological regulation pathways, including organ development, cell differentiation, proliferation and apoptosis (12,13). Furthermore, increasing evidence indicates that epigenetic perturbations may contribute to abnormal miRNA expression in cancer cells (14). Aberrant miRNA expression has been reported in a variety of human solid tumors cancers, including lung, breast, liver, thyroid and ovarian cancer (15–20). Therefore, expression level alterations may commonly occur in non-coding miRNAs, resulting in a series of tumor behaviors that are typical of EEC.

Numerous studies have reported the expression profiles of tissue miRNAs in EEC (21–25). Our study group has previously systematically discovered that the unique expression patterns of these circulating miRNAs are correlated with certain human diseases (26–29). The process of collecting tissue samples is invasive, as it relies on surgical sections. In contrast, serum samples may be obtained with easier accessibility and handled with lower cost. The miRNA fingerprint of gynecological malignancies remains in its infancy. To date, no existing data has described the global miRNA pattern in EEC sera.

The present study investigated the miRNA expression profiles in the serum of patients with EEC, and attempted to identify the miRNAs that were capable of functioning as novel and minimally invasive clinical biomarkers for this gynecologic malignancy.

## Patients and methods

### Study design, patients and controls

**Figure 1.**
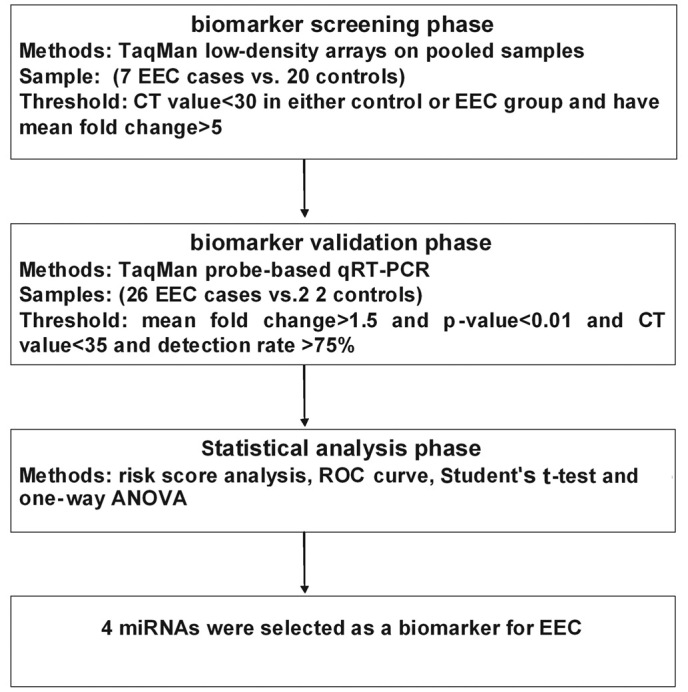
A flow chart of the experimental design. ECC, endometrioid endometrial cancer; qRT-PCR, quantitative reverse transcription polymerase chain reaction; ROC, receiver operating characteristic.

Approval for the present study was obtained from the ethics committee of each participating institution and all samples were collected from consenting individuals. The study design that was used to identify a serum miRNA profile for EEC is shown in Fig. 1. A total of 33 patients with primary endometrial cancer and 42 healthy control subjects were enrolled in the study. During the initial screening stage, the EEC serum samples pooled from seven EEC patients and the control samples pooled from 20 normal donors were subjected to TaqMan^®^ low-density arrays (TLDAs; Invitrogen, Carlsbad, CA, USA) to identify the miRNAs that were significantly differentially expressed. Subsequently, a confirmation analysis was performed using a hydrolysis probe-based stem-loop quantitative reverse transcription polymerase chain reaction (qRT-PCR) assay to refine the number of serum miRNAs that were used as an EEC signature. All patients had been diagnosed with EEC and were treated at Jinling Hospital (Nanjing University, Nanjing, Jiangsu, China) between March 2010 and December 2011. Blood samples were collected prior to any therapeutic procedures, including surgery, chemotherapy and radiotherapy. The histopathology results were confirmed by surgical resection of the tumors, and the tumor stage was defined according to the International Federation of Gynecology and Obstetrics (FIGO) system criteria (4). For the patients who were unsuitable for surgical management, the histopathology characteristics and tumor stages were confirmed using a histobiopsy and imaging technology. The control participants were recruited from a large pool of individuals seeking a routine health checkup at the Jinling Hospital.

The demographics and clinical features of the patients in the validation set are listed in Table I. The controls were matched to the patients by age, sex and ethnicity. None of the healthy controls had previously been diagnosed with malignancies.

### Sample processing and RNA extraction

For the TLDA of the serum, an equal volume of serum from each participant was pooled separately to form patient and control sample pools. TRIzol reagent (Invitrogen) was used to extract the total RNA from each pool of the serum samples (12.5 ml/sample). The aqueous phase was subjected to 3 steps of acid phenol/chloroform purification in order to eliminate the protein residues prior to the isopropyl alcohol precipitation. The resulting RNA pellet was dissolved in 20 *μ*l RNase-free water and stored at −70°C until further analysis.

For the qRT-PCR assay, the total RNA was extracted from 100 *μ*l serum with a 1-step phenol/chloroform purification procedure. A solution containing 100 *μ*l serum, 200 *μ*l acid phenol, 200 *μ*l chloroform and 300 *μ*l diethylpyrocarbonate-treated water was vortex-mixed vigorously and incubated at room temperature for 15 min. Following phase separation, the aqueous layer was mixed with 40 *μ*l sodium acetate (3 mol/l, pH 5.3) and 800 *μ*l isopropyl alcohol. This solution was stored at −20°C for 1 h. The RNA pellet was collected by centrifugation at 16,000 x g for 20 min at 4°C. The resulting RNA pellet was washed once with 750 ml/l ethanol and dried for 10 min at room temperature. Finally, the pellet was dissolved in 20 *μ*l ribonuclease-free water and stored at −70°C until further analysis.

### TaqMan low density arrays of serum miRNAs

RT was performed using the TaqMan microRNA RT kit and Megaplex RT primers (Invitrogen). Briefly, total RNA (3 *μ*l) was added to 4.5 *μ*l RT reaction mix, which consisted of 10X Megaplex RT primers, dNTPs with 100 mM dTTP, 50 U/*μ*l MultiScribe reverse transcriptase, 10X RT Buffer, 25 mM MgCl_2_, 20 U/*μ*l RNase inhibitor and nuclease-free water. Following incubation on ice for 5 min, RT was performed using a thermal cycler (UNO-Thermoblock; Biometra, Göttingen, Germany). To increase the sensitivity of the TLDA, a pre-amplification was performed following the RT procedure. MicroRNA profiling of 754 varying human mRNAs was then performed using the TLDAs (TaqMan array human microRNA A+B cards set v3.0; Invitrogen). All reactions were performed according to the manufacturer’s instructions. All steps were performed using a 7900HT Fast Real-Time PCR System (Applied biosystems, Foster City, CA, USA). The results were expressed as Ct values and normalized on the calculated median Ct of each sample (ΔCt). Relative expression was calculated using the comparative Ct method (2^−^^ΔΔCt^). U6 was used as an internal reference.

### Quantitative RT-PCR

The total RNA (2 *μ*l) was reverse transcribed to cDNA using AMV reverse transcriptase (Takara Dalian, Liaoning, China) and the stem-loop RT primer (Applied Biosystems). Real-time PCR was performed using TaqMan miRNA probes (Applied Biosystems) on the Applied Biosystems 7300 Sequence Detection System. All reactions were run in triplicate. Following the reaction, the Ct values were determined using the fixed threshold settings. The absolute concentrations of the target miRNAs were calculated using calibration curves, which were developed with corresponding synthetic miRNA oligonucleotides (Takara) of known concentrations (1–10^6^ fM/l).

### CA-125 determination

The content of the serum and the CA-125 level were measured by chemiluminescence immunoassay using an ARCHITECT™ i2000SR access immunoassay system (Abott; Lake Forest, IL, USA).

### Statistical analysis

The quantitative data are presented as mean ± standard error. The qRT-PCR was performed in triplicate for three or more independent experiments. The statistical significance was determined using the Student’s t-test, and P<0.05 was considered to indicate a statistically significant difference. For each miRNA, a ROC curve was constructed and the area under the curve (AUC) was calculated in order to evaluate the specificity and sensitivity of the endometrial cancer prediction. A risk score analysis was performed to evaluate the association between cervical cancer and the serum miRNA expression levels.

The risk score of each miRNA (s), was set to 1 if the expression level was greater than the upper 95% reference interval for the corresponding miRNA level in the controls, and to 0 otherwise. A risk score function (RSF) to predict the endometrial cancer risk was defined according to a linear combination of the expression level for each miRNA. For example, the RSF for sample i using information from four miRNAs was:

$${\text{RSF}}_{i}=\Sigma_{j=1}^{4}W_{j}\cdot s_{\mathrm{ij}}$$

In this equation, sij represents the risk score for miRNA j on sample i, and Wj is the weight of the risk score of miRNA j. To determine the Ws, five univariate logistic regression models were fitted using the disease status with each of the risk scores. The regression coefficient of each risk score was used as the weight to indicate the contribution of each miRNA to the RSF. Frequency tables and ROC curves were then used to evaluate the diagnostic effects of the profiling and to identify the appropriate cut-off point. All statistical analyses were performed using the Statistical Analysis System software (version 9.1.3; SAS Institute, Cary, NC, USA).

## Results

### Patient description

During the initial biomarker screening stage, the serum samples from seven EEC cases and 20 matched controls were subjected to TLDA. The significantly altered miRNAs were selected and validated in an additional 26 EEC patients and 22 controls. All 33 patients that were enrolled in the present study had a clinical and pathological diagnosis of EEC. No significant difference was observed in the distribution of age or marital or menopausal status between the cancer patients and the normal subjects. In general, the EEC patients and control subjects had no other diseases, including significant cardiac dysfunction, active infections (hepatitis or tuberculosis), neurological diseases or diabetes at the time that the blood was drawn (Table I).

### Distinct miRNA signatures in EEC compared with controls observed by TLDA

In the biomarker screening phase, the miRNA expression profile of 754 miRNAs were determined in the serum from the seven endometrial cancer patients using TLDA technology, and were then compared with the expression profiles of the 20 healthy individuals. In the EEC patients, 123 miRNAs (fold change, FC≥1.5) were upregulated, 24 miRNAs (FC<0.5) were downregulated and 80 miRNAs were expressed at similar levels. The remaining 527 miRNAs were not detected in the serum. The criteria for further investigation of the most promising candidates were as follows: i) high mRNA levels in the serum of the cancer patients, FC≥5 and ii) quantification cycle values of 30 to enable reliable detection. Based on these criteria, 22 miRNAs were identified to be differentially expressed in EEC and were further analyzed using qRT-PCR (Table II).

### Evaluation of miRNA expression by real-time qRT-PCR analysis

**Figure 2.**
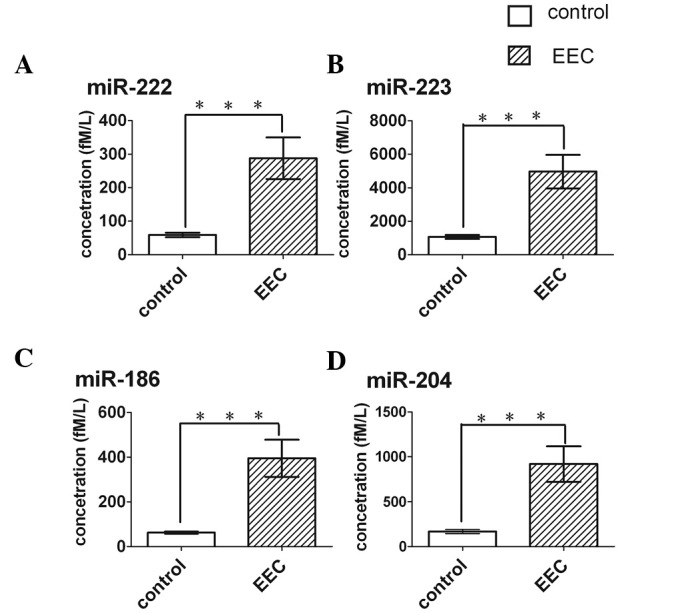
Detection of EEC using the 4-serum miRNA profile as a biomarker. (A–D) Serum levels of the four miRNAs were measured in 26 EEC cases and 22 healthy control subjects (in the validation set) using a hydrolysis probe-based qRT-PCR assay (^***^P<0.001). qRT-PCR, quantitative reverse transcription polymerase chain reaction; EEC, endometrioid endometrial cancer.

A qRT-PCR assay was used to confirm the expression of the candidate miRNAs that were selected from the previous step. Semi-logarithmic plots of the calibration curves for various concentrations of the synthetic single-stranded miRNA calibrators were linear from 10 fmol/l to 10^4^ pmol/l. The standard curves of miR-222, miR-223, miR-186 and miR-204, created using synthetic miRNAs were as follows: y = −3.468 x +34.238, R^2^=0.9976; y = −3.853 x +36.188, R^2^=0.9964; y = −3.2448 x +33.736, R2=0.9972; and y = −3.389 x +35.454, R^2^=0.9951. In this phase, only the miRNAs with a mean FC of ≥1.5 and with P<0.01 were selected. On a follow-up qRT-PCR of the candidate miRNAs, the levels of four miRNAs were significantly higher (miRNA-222, -223, -186, and -204; P=6.3, 3.2, 2.6 and 4.5×10^−4^, respectively) in the serum of the endometrial cancer patients compared with the control subjects. When compared with the miRNA expression in the normal controls, the FCs of the EEC patients were 4.87, 4.35, 5.93 and 5.21, respectively. Fig. 2 shows the various concentrations of the miRNAs in the cancer patients compared with the control subjects. During the analysis and validation phase, a profile of the four serum miRNAs was generated, which served as a potential biomarker for EEC for the next analysis.

### Risk score and ROC curve analysis

**Figure 3.**
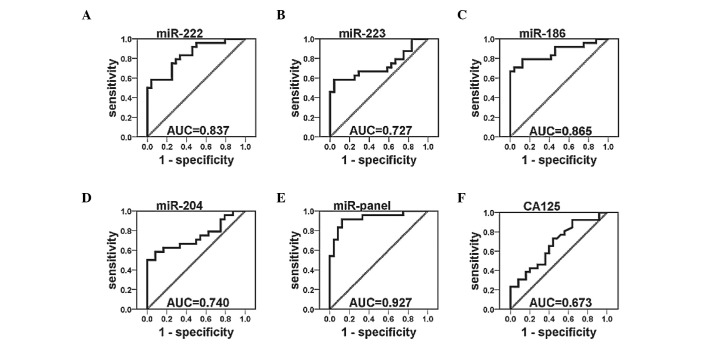
(A–D) ROC curves for the abilities of the serum concentations for the 4 individual microRNAs (miRNAs). (E) The four-miRNA panel and (F) carbohydrate antigen 125 (CA-125) to differentiate the EEC cases (n=26) from the controls (n=22). ROC, receiver operating characteristic; EEC, endometrioid endometrial cancer; AUC, area under the curve.

The ROC curves, which were constructed to compare the relative concentrations of the four miRNAs in the EEC patients with those in the healthy controls, yielded the following AUCs: miR-222, 0.837 (95% CI, 0.726–0.948); miR-223, 0.727 (95% CI, 0.577–0.877); miR-186, 0.865 (95% CI, 0.755–0.974); and miR-204, 0.740 (95% CI, 0.594–0.885; Fig. 3A–D). From the four miRNAs investigated, miR-186 displayed the highest sensitivity and specificity for diagnosing endometrial cancer (Fig. 3B)

For the further evaluation of the diagnostic value of the four-miRNA profiling system, a risk score formula was used to calculate the RSF for the EEC and control samples. The samples were ranked according to their RSF and then divided into a high-risk group representing the predicted EEC cases and a low-risk group representing the control individuals. The frequency table and ROC curves were then used to evaluate the diagnostic effect of the four-miRNA profiling system and to identify the appropriate cut-off point. The subsequent addition of each of the four miRNAs was able to incrementally improve the stratification power characterized with an AUC of 0.927 (95% CI, 0.845–1.000; Fig. 3E). With an optimal cut-off value, in which the sum of the sensitivity and specificity was maximal, the specificity was 87.5% and the sensitivity was 91.7%. In contrast, the AUC value for CA-125 was a much lower value of 0.673 (95% CI, 0.525–0.821; Fig. 3F). These results indicate that the four-miRNA signature is a more reliable and accurate method of diagnosing EEC compared with the single miRNA-based assay and CA-125.

## Discussion

In the present study, four serum miRNAs (miR-222, miR-223, miR-186 and miR-204) were observed to be significantly upregulated in the endometrial cancer patients compared with the control subjects, and may serve as a non-invasive, accurate biomarker for the diagnosis of EEC. To the best of our knowledge, this is the first study to identify a serum miRNA-based EEC signature using a genome-wide serum miRNA expression profiling analysis.

Serum-based biomarkers allow the composite analysis of tumors without using invasive procedures, including biopsies or surgery. The reasons that circulating miRNAs have an enormous potential to serve as an ideal class of cancer biomarkers are as follows: i) miRNA expression is known to be aberrant in cancer (30–32), and tumor cell-derived miRNAs in circulation may be stored in microvesicles that are secreted by various cell types (33–35); ii) miRNA expression profiles are pathognomonic or tissue-specific (31); iii) miRNAs are remarkably stable molecules that have been shown to be well preserved by serum and other body fluids (33–35), and are readily detected by various assays, such as the qRT-PCR assay, a widely used technique in clinical laboratories (33). miRNAs may passively leak from apoptotic or broken cells, or be actively secreted by cell-derived microvesicles (MVs) or as MV-free miRNAs, with the latter considered to be a major source of serum and plasma miRNA (12,36).

The early studies on the search for serum miRNA-based cancer biomarkers generally focused on individual cancer-specific miRNAs (37). However, the diverse and complex molecular events that are involved in the initiation and development of a malignancy makes the use of individual miRNAs as tumor biomarkers lack credibility. Therefore, although one particular miRNA in serum alone may help to distinguish between patients and healthy controls, a panel of miRNAs has a greater potential to offer a more specific diagnosis. In the validation phase of the present study, four candidate miRNAs (miR-222, miR-223, miR-186 and miR-204) were tested in an independent cohort of endometrial cancer patients. The overexpression of the four miRNAs in the serum of the endometrial cancer patients compared with the healthy controls reached statistical significance with P<0.001. The combination of serum miR-222, miR-223, miR-186 and miR-204 levels yielded a specificity of 87.5% and a sensitivity of 91.7%, and proved to be an even more powerful discrimination tool. The results clearly demonstrate that a combination of multiple serum miRNAs is a more comprehensive indicator for tumor detection than the conventional single protein-based or carbohydrate molecule-based biomarkers.

Cell-secreted miRNAs are highly stable and may serve as biomarkers for various diseases and signaling molecules in intercellular communication. However, the mechanism underlying the stability of circulating miRNAs is not well understood. The functional study of candidate miRNAs in tumor tissues and their target proteins may provide additional evidence supporting the use of serum miRNAs as reliable diagnostic biomarkers. Upregulated miR-222 has been observed in prostate cancer (38), primary glioblastoma (39), papillary thyroid carcinoma (19) and breast cancer (40), and a possible functional role has been proposed for miR-222 in cell growth and proliferation due to its effect on the expression of cell cycle regulatory proteins, including the cyclin-dependent kinase inhibitor p27Kip1 (40–42). miR-223 is upregulated in the tissue samples of certain digestive system neoplasms, including gastric cancer, colon cancer and pancreatic cancer (43,44). Wu *et al* revealed that miR-223 regulates FOXO1 expression and cell proliferation (45). miR-186 has been shown to be significantly upregulated in endometrial cancer compared with the normal endometrium, and the expression of miR-186 was observed to be sufficient enough to significantly reduce the abundance of FOXO1 (46). Chung *et al* observed that the dysregulation of miR-204 mediates the migration and invasion of endometrial cancer by regulating FOXC1 (30). In summary, three miRNAs from the signature profile identified in the present study are closely linked to the expression of the FOX proteins. FOX family members, including FOXA1, FOXC1, FOXO1 and FOXP1, were identified to be aberrantly expressed in EEC, and their dysregulation may contribute to carcinogenesis, including metastasis (47–49). The association between miRNAs and the FOX family members strengthens the observation that the serum miRNA profile serves as a tumor fingerprint for EEC.

The strengths of the present study are that the four-miRNA signature that was identified displays a marked difference between patients and healthy controls, and that a combination of miRNAs has the potential to offer more sensitive and specific diagnostic tests. Although the observations are promising, further validation using a larger number of patient samples or an additional correlation analysis between miRNA signatures and long-term patient outcome is required.

In conclusion, the expression profiles of four serum miRNAs have been demonstrated to serve as non-invasive biomarkers for EEC detection. This observation will trigger interest in further intensive research into the elucidation of their functional effects, so as to improve our knowledge with regard to the role that these novel biomarkers play in carcinogenesis, and to expose their true potential as therapeutic agents.

## Figures and Tables

**Table I.** Demographic and clinical characteristics of the patients and control individuals in the validation phase.

Variables	Cases (n=26)	Controls (n=22)	P-value
Average age (years)^a^			
Mean (SD)	55.5 (7.2)	54.7 (9.1)	0.912^a^
≥55years, n (%)	16 (61.5)	15 (68.2)	0.860^b^
<55years, n (5)	10 (38.5)	7 (31.8)	
Marital status, n (%)			
Married	26 (100.0)	21 (95.5)	0.933^b^
Unmarried	0 (0.0)	1 (4.5)	
Menopausal status, n (%)			
Postmenopausal	19 (73.1)	18 (81.8)	0.709^b^
Premenopausal	7 (26.9)	4 (18.2)	
FIGO stage, n (%)			
I	23 (88.5)		
II	3 (11.5)		
Histological grade, n (%)			
Moderately or poorly	21 (80.8)		
Well-differentiated	5 (19.2)		
Significant cardiac dysfunction, n (%)			
Yes	2 (7.7)	0 (0.0)	0.546^b^
No	24 (92.3)	22 (100.0)	
Neurological disease or diabetes, n (%)			
Yes	4 (15.4)	1 (4.5)	0.453^b^
No	22 (84.6)	21 (95.5)	

a Student’s t-test,

b Two-sided χ^2^ test. FIGO, International Federation of Gynecology and Obstetrics.

**Table II.** Differentially-expressed miRNAs in EEC serum samples compared to controls determined by TLDAs.

miRNA	Controls (Ct value)	EEC patients (Ct value)	FC
hsa-miR-150	21.99	14.89	257.76
hsa-miR-223	20.89	15.03	109.20
hsa-miR-195	26.95	21.94	60.53
hsa-miR-202	28.97	23.99	58.90
hsa-miR-126	26.99	22.60	39.61
hsa-miR-342-3p	23.98	19.77	34.83
hsa-miR-25	26.96	22.94	30.78
hsa-miR-19b	25.96	22.32	23.44
hsa-miR-16	22.96	19.44	21.58
hsa-miR-222	23.96	20.61	19.16
hsa-miR-191	22.93	19.62	18.64
hsa-miR-106a	25.99	22.93	15.59
hsa-miR-601	24.95	21.95	15.02
hsa-miR-17	25.99	23.38	11.48
hsa-miR-186	27.98	25.39	11.39
hsa-miR-26b	26.97	24.53	10.22
hsa-miR-155	27.97	25.71	9.01
hsa-miR-92a	23.98	21.96	7.67
hsa-miR-30c	24.96	22.97	7.46
hsa-miR-139-5p	27.93	26.07	6.85
hsa-miR-24	23.98	22.27	6.12
hsa-miR-204	28.93	27.31	5.79

TLDAs, TaqMan low-density arrays; EEC, endometrioid endometrial cancer; FC, fold change; miRNA, microRNA.
